# Infantile Spasms Treated with Intravenous Methypredinsolone Pulse

**Published:** 2017

**Authors:** Afagh HASSANZADEH RAD, Vahid AMINZADEH

**Affiliations:** 1 Pediatrics Growth Disorders Research Center, School of Medicine, 17th Shahrivar Hospital, Guilan University of Medical Sciences, Rasht, Iran

**Keywords:** Infantile spasms, Prednisolone, Administration, Intravenous

## Abstract

**Objective:**

Infantile spasms is diagnosed late even by expert pediatricians. Late diagnosis (later than 3 weeks) can have a negative effect on the long-term prognosis. We aimed to investigate infantile spasms treated with intravenous methylprednisolone pulse.

**Materials & Methods:**

In this case series study, 20 infants with infantile spasms in 17-Shahrivar Hospital, Rasht, Iran were enrolled. Drugs were administered based on Mytinger protocol that included 3 days of methylprednisolone pulse and 56 days of oral prednisolone. The control of spasms and the omission of hypsarrhythmia in infants follow-up were the primary and secondary outcomes, respectively. Remission was indicated if the caregivers mentioned no spasms or >50% decrease regarding drug initiation for at least 5 consecutive days and the electroencephalography during sleep period noted the omission of hypsarrhythmia.

**Results:**

Eleven female (55%) and 9 male (45%) patients with the mean age of 4.95±1.39 months were enrolled. Mean rapid remission was noted as 4.41±1.50 days. Twelve patients (60%) noted early remission. seizure was controlled in 3(15%) patients completely after 24 months. Five (25%) occasional seizures were noted controlled by routine anticonvulsant drugs after 24 months and 12 (60%) no response was mentioned. Most of the patients (65%) had cryptogenic etiology for infantile spasms. Uncontrolled seizure was mentioned after initial remission.

**Conclusion:**

Methyl prednisolone is an appropriate drug based on easy administering, low cost, and its accessibility.

## Introduction

Infantile spasms (IS) is an age-dependent myoclonic seizure that occurs in 25 per 100000 live births. Its etiology can’t be defined in 75% of patients with infantile spasms. It usually initiates in 4-8 months after birth (the peak: 4-7 months) and always before 1 year of age (1).

According to the international league against epilepsy, infantile spasms are classified into symptomatic and cryptogenic types. Commonly, there is a sign of previous brain injury or a related to a specific etiology in symptomatic type. However, in cryptogenic type, no sign of brain injury or specific etiology can be noted. As infantile spasms is a medical emergency, early diagnosis especially for cryptogenic type is mandatory (2).

Generally, infantile spasms diagnoses late even by expert pediatricians and late diagnosis (later than 3 wk) can have a negative effect on the long-term prognosis (3). It is generally distinguished with common infantile disorders such as infantile colic. Electroencephalography is the most important method of diagnosis of infantile spasms showed by hypsarrhythmia (1).

Although the ultimate development of infants depends on the cause but most of them encounter with diverse types of seizures and psychomotor retardation (4).

There is no specific treatment preferred in contrast with other types of treatment (5). American Academy of Neurology and pediatrics neurology society mentioned that there is no document that successful treatment can affect late prognosis. ACTH can be noted as a shortterm treatment for infantile spasms, and vigabatrin can be effective (2). Although, ACTH is the traditional treatment of IS, however, oral prednisolone with the dosage of 2-4 mg/ kg/d for 8 wk can be an alternative method (1). Generally, 25%-33% of recurrence occurs after any type of treatment (6). Although, the preference of ACTH was reported on prednisolone but that can be effective when no response is reported by administering ACTH and vice versa (7).

Worldwide, ACTH is an expensive drug with a low access (scarce access), therefore, according to safety, cheap and easily accessible of intravenous methylprednisolone (8,9), we aimed to investigate the effect of intravenous methylprednisolone pulse on the infantile spasms in patients hospitalized in 17-Shahrivar Hospital, Rasht, Iran.

## Materials & Methods

This observational case series study was conducted on 20 infants with IS hospitalized in 17 Shahrivar Hospital, Rasht, Iran during 2011-2014. Infants aged less than 1 year and more than 1 month with any etiology of spasms except tuberous sclerosis (vigabatrin is their choice treatment) (3), symptom of spasms and Hypsarrhythmia in EEG were included. Patients with kidney disease, hypertension, cardiac disease, and febrile or infectious diseases were excluded.

Consent letters were obtained from parents and the study was approved by Ethics Committee of the hospital.

Spasms were classified into cryptogenic and symptomatic types based on history, physical exam, brain MRI, and metabolic study. Patients were hospitalized in Neurology Ward to treat with methylprednisolone pulse. 

Vital signs were checked and parents were educated. In addition, fasting blood sugar, blood pressure and urine analysis and culture were monitored. Drugs were administered based on Mytinger protocol that included 3 days of methylprednisolone pulse and 56 days of oral prednisolone (Table 1) (10).

Primary outcome was the control of spasms and the second outcome was the omission of hypsarrhythmia in infants follow-up. Although prolonged video EEG is the most appropriate method for defining response but if there is no access, clinicians can consider routine EEG as well. Response was indicated if the caregivers mentioned no spasms or >50 % decrease regarding drug initiation for at least 5 consecutive days and the EEG during sleep period noted the omission of hypesarythmia (6). The minimum period of response was 2 wk (6, 10).

The mentioned protocol will be used as a first treatment or will be added to the previous ineffective drugs with no change in the previous dosage of drugs (11).

Patients were followed up for 24 months after treatment. Data were gathered by a form including age, sex, etiology, previous treatment, period of spasms control, follow-up, EEG, and complications. Data were reported by number, percent, mean and standard deviation by SPSS software version 19.0 (Chicago, IL, USA).

## Results

Eleven female (55%) and 9 male (45%) patients with the mean age of 4.95±1.39 months participated in this study. Mean rapid remission was noted as 4.41±1.50 days. Tweleve patients (60%) noted that early remission. Seizure was controlled in 3 (15%) patients completely after 24 months. Five (25%) occasional seizures were noted controlled by routine anticonvulsant drugs after 24 months and 12 (60%) no response was mentioned. 

At the follow-up EEG, 13 patients (65%) were normal and 7 (35%) abnormal cases were noted. Most of the patients (65%) had cryptogenic etiology for infantile spasms. Seven (35%) repeated seizures, 5 (25%) occasional seizures, 2 (10%) refractory seizures and 3 (15%) uncontrolled seizure were mentioned after initial remission. Besides, no seizure was noted in 3 (15%) patients after initial remission. Seven patients (35%) received only methylprednisone and ethylprednisolone was added to previous drugs in 13 patients. No complication was noted in most of the patients (13 cases). However, 5-weight gain, one asymptomatic glucosuria, and 1 irritability were noted in patients with infantile spasms (Table 2).

**Table 1:** Protocol for intravenous methylprednisolone with oral prednisolone

**Medication**	**Time period**	**Dose (mg/kg)**
Intravenous methylprednisolone	Day 1-3	20/d
Oral prednisolone	Day 4-17 (2 weeks) Day 18-31 (2 weeks) Day 32-45 (2 weeks) Day 46-52 (1 week) Day 53-59 (1 week)	4/d 3/d 2/d 2/every other day 1/every other day

**Table 2:** Patient Characteristics and Outcomes

**num**	**Age** **(months)** **)**	**sex**	**Etiology**	**Interval** **Between onset of spasm and treatment (day)**	**Early remission (day)**	**Follow up EEG**	**treatment**	**Late remission**	**complication**
**1**	3	F	Cryptogenic	5	3	Abnormal (hypes arythmia)	MP	No seizure	Weight gain
**2**	4	M	Congenital CMV	15	No response	Normal	MP+PB+NZP	Uncontrolled seizure	Irritability
**3**	5	F	PKU	21	2	Abnormal	MP+TPM+PB	Occasional seizure	-
**4**	7	M	Cryptogenic	60	No response	Normal	MP+PB+CZP	Uncontrolled seizure	-
**5**	4	F	Structural	12	4	Normal	MP	Occasional seizure	-
**6**	3	F	Cryptogenic	8	6	Normal	MP+PB+CLB	No seizure	Weight gain
**7**	6	F	Cryptogenic	30	No response	Abnormal	MP+PB	Repeater seizure	-
**8**	5	M	Hypoxic ischemic encephalopathy	12	No response	Normal	MP+CZP+PB	Repeated seizure	-
**9**	3	F	Cryptogenic	8	5	Normal	MP+PB+NZP	No seizure	Weight gain
**10**	5.5	M	Cryptogenic	17	3	Normal	MP+PB+NZP	Occasional seizure	-
**11**	5	M	Cryptogenic	14	5	Abnormal	MP+PB+TPM	Occasional seizure	Weight gain
**12**	6.5	M	Structural	20	No response	Normal	MP	Refractory seizure	-
**13**	3.5	F	CMV	10	4	Normal	MP+PB+TPM+LGM	Occasional seizure	-
**14**	5	M	Cryptogenic	10	3	Abnormal	MP+PB+CZP	Repeated seizure	-
**15**	8	F	Cryptogenic	60	No response	Normal	MP	Refractory seizure	Asymptomatic glucosuria
**16**	5	M	Cryptogenic	12	6	Normal	MP+PB+TPM	Repeated seizure	-
**17**	4.5	F	Down syndrome	17	5	Abnormal	MP+PB+NZP	Repeated seizure	-
**18**	7	M	Cryptogenic	21	No response	Normal	MP	Repeated seizure	Weight gain
**19**	5	M	structural	15	7	Normal	MP	Repeated seizure	-
**20**	5	F	Cryptogenic	10	No response	Normal	MP+CZP+LGM	Repeated seizure	-

## Discussion

According to results, 60% of patients noted remission which was consistent with Mytinger et al. They also indicated 50% remission after administering pulse methyl predinsolone.(10) Azam et al compared remission by administering ACTH (33 patients) and oral predinsolone(72 patients) in 105 infants with infantile spasms. They mentioned improvement in 27 patients who received ACTH. From these 27 patients, 11 remained spasm free. 51 patients in oral prednisolone group responded and 17 remained spasm free after cessation the drug. Also, administering high dose oral predinsolone in patients with infantile spasms indicated 67% spasm free during 2 weeks. (11)LUX et al reported 54%, 76%, and 73% improvement by administering vigabatrin, ACTH and oral predisolone in 208 patients with infantile spasms (12). 

In this study mean rapid remission was mentioned as 4.41±1.50 days which was similar with previous investigation. They noted rapid remission in 2-6 days(10).

At the follow up, 13 patients(65%) were normal and 7 (35%)abnormal cases were noted. However, mytinger et al noted lower rate of normal results(50%)(10). 

Although our results indicated no complication in most of the patients by administering pulse methylpredinsolone. 

But mytinger et al indicated asymptomatic hypertension and probable adrenal insufficiency in patients treated with pulse methyl predinsolone. LUX et al noted 19 complications including gasterointestinal complication, irritability, drowsiness, infection, increased appetite, dermatological complication, mood disturbance, and neuropsychiatric disorders by administering oral predinsolone. However, they noted lower complications by administering ACTH and vigabatrin (12). Kossoff et al noted 53% complications in patients received oral predinsolone including irritability, edema, weight gain and hypertension (13).

As clinicians hospitalize patients for a short period of time (3 days) to administer pulse methyl predinsole and during hospitalization, short term Complications of the pulse can be detected directly by clinicians and parents can be completely educated for follow up; Investigators proffered it versus oral type. Also, patients hospitalized can be better evaluated before treatment especially for asymptomatic infections and emergently clinicians can manage probable complications, it seems that Pulse methyl predinsolone can be recommended as a choice of treatment. Regarding similar effects of pulse methyl predinsolone in comparison with ACTH, it seems that methyl predinsolone is a preferable drug regarding easy administering, low cost and its accessibility. 

## Author`s Contribution

Hassanzadeh Rad A: Interpretation of data for the work, drafting the work or revising it critically for important intellectual content, Final approval of the version to be published, Agreement to be accountable for all aspects of the work in ensuring that questions related to the accuracy or integrity of any part of the work are appropriately investigated and resolved Aminzadeh V: Substantial contributions to the conception or design of the work; or the acquisition, analysis, or interpretation of data for the work, drafting the work or revising it critically for important intellectual content, Final approval of the version to be published, Agreement to be accountable for all aspects of the work in ensuring that questions related to the accuracy or integrity of any part of the work are appropriately investigated and resolved.

## Conflict of interest

The authors declare that there is no conflict of interest.
